# Treatment Approaches for Melanomas That Relapse After Adjuvant or Neoadjuvant Therapy

**DOI:** 10.1007/s11912-022-01288-y

**Published:** 2022-05-31

**Authors:** Gary Ng, Wen Xu, Victoria Atkinson

**Affiliations:** 1 Princess Alexandra Hospital, Brisbane, Australia; 2 University of Queensland, Brisbane, Australia; 3 Greenslopes Private Hospital, Brisbane, Australia

**Keywords:** Melanoma, Adjuvant, Neoadjuvant, Immunotherapy, Targeted therapy, PD-1, BRAF, Relapse, Recurrence

## Abstract

**Purpose of Review:**

Effective adjuvant treatment with immunotherapy and targeted therapy has significantly improved outcomes for patients with resectable locally advanced or metastatic melanoma, but a substantial proportion unfortunately relapse. Here, we review available data and explore evolving research which might impact decision-making in this setting.

**Recent Findings:**

Small retrospective studies have explored pattern of disease relapse and observed outcomes of subsequent treatment. There are ongoing trials in the neoadjuvant setting which may provide valuable information regarding disease response and potentially change the way we approach disease relapse.

**Summary:**

Currently there is limited evidence to guide clinicians in managing melanomas that relapse after adjuvant therapy. Standardised data collection and future prospective studies are needed.

## Introduction

Patients who have undergone adequate surgical excision of melanoma with locoregional disease remain at a high risk of disease relapse and death. In the latest 8th edition of the American Joint Committee on Cancer (AJCC) staging system, patients with clinically detectable nodal or in-transit disease, namely stage IIIB, IIIC and IIID had a 10-year survival of 77%, 60% and 24% respectively [1]. The development of effective adjuvant systemic treatment options has significantly altered the treatment landscape in managing these patients; despite this, a significant proportion will relapse. In these situations, there is little data to guide oncologists on the most effective therapeutic options.

Historically, patients with resected stage III disease had limited options, namely adjuvant interferon-alfa, radiotherapy or observation alone. Interferon-alfa was the first systemic treatment approved for the management of resected stage III melanoma but was never a widely accepted standard of care due to significant toxicity including fatigue, flu-like symptoms, depression including suicidal ideation, myelotoxicity, hepatotoxicity and autoimmune disease [2]. While a marginal benefit for relapse-free survival (RFS) was demonstrated, a benefit for overall survival (OS) could not be consistently demonstrated with conflicting trial results, although a meta-analysis showed a marginal survival benefit of approximately 3% [3].

The benefit of adjuvant radiotherapy was also limited, with one randomised control trial showing a reduced risk of recurrence in the nodal field following lymph node dissection, but no survival benefit compared to observation alone [4]. Long-term toxicities include persistent pain, radiation skin changes, scarring, telangiectasia and lymphoedema.

The ‘modern’ era of adjuvant treatment was heralded by the development of checkpoint inhibitor immunotherapy and BRAF-targeted therapy for advanced disease. The first ‘modern’ phase III adjuvant immunotherapy trial for stage III resected melanoma was initially reported in 2015, demonstrating the efficacy of ipilimumab, followed by three landmark trials in 2017–2018 showing significant benefit with nivolumab, dabrafenib/trametinib and pembrolizumab [5–7].

Although follow-up was relatively early at initial reporting with a current lack of mature OS data, the impressive RFS benefit and favourable tolerability for all three treatment options have been convincing enough for these agents to become standard of care in the adjuvant setting.

Despite the proven RFS benefit, a significant proportion of patients will develop disease relapse and subsequent management for these patients remains undefined by the clinical trial data. This review will discuss the available data and considerations for patients that relapse on or after adjuvant immunotherapy or targeted therapy.

## Adjuvant Immunotherapy

Although ipilimumab remains the sole adjuvant immunotherapy agent with mature overall survival data, it has never been widely embraced as standard of care due to toxicity concerns. The phase III EORTC 18071 trial compared ipilimumab (10 mg/kg) with placebo for patients with resected stage III (AJCC 7th edition [AJCCv7]) cutaneous melanoma and found a significant improvement in RFS and OS. Due to immune related adverse events, 40% of patients did not make it to maintenance dosing and the rate of grade ≥ 3 toxicity was 54% with 5 treatment-related deaths [8]. A subsequent phase III trial (E1609) assigned patients with resected stage IIIB to IVM1b disease (AJCCv7) to ipilimumab at 10 or 3 mg/kg or high-dose interferon alfa-2b (HDI). Ipilimumab at 3 mg/kg compared with HDI showed an improved 5-year OS of 72% versus 67% (hazard ratio (HR) 0.78, 95% confidence interval (CI) 0.61–0.99, *p*-value 0.044), and the benefit of ipilimumab at 10 mg/kg over HDI did not reach statistical significance. The rate of grade ≥ 3 toxicity was 37% in the ipilimumab 3 mg/kg group compared with 58% in the 10 mg/kg group [9].

These results, however, were superseded with the use of adjuvant anti-programmed death 1 (anti-PD1) antibodies nivolumab and pembrolizumab, which have shown improved RFS compared to ipilimumab and placebo in phase III trials, respectively, and with a much more favourable safety profile.

In CheckMate 238, nivolumab showed superior RFS compared with high-dose ipilimumab (10 mg/kg) in patients with resected stage IIIB, IIIC and IV disease (AJCCv7) [6, 10]. After a median follow-up of 51 months, 4-year RFS was 51.7% in the nivolumab group and 41.2% in the ipilimumab group (HR 0.71, 95% CI 0.60–0.86, *p*-value 0.0003). The 4-year OS rates were similar at 77.9% and 76.6%. However, nivolumab was much better tolerated, with a grade ≥ 3 toxicity rate of 14.4% compared with 45.9% with ipilimumab.

Pembrolizumab was compared with placebo in KEYNOTE-054 and included patients with stage IIIA (metastasis > 1 mm), IIIB and IIIC disease (AJCCv7) [7, 11]. Of note, patients on the placebo arm were permitted to crossover at recurrence and receive pembrolizumab for up to 24 months. In the 3-year analysis in the intention-to-treat population, RFS was 63.7% in the pembrolizumab group and 44.1% in the placebo group (HR 0.56, 95% CI 0.47–0.68, *p*-value < 0.001). The rate of grade ≥ 3 adverse events in the pembrolizumab group was 14.5%.

It was hoped the crossover design of KEYNOTE-054 will answer the question of upfront adjuvant therapy versus therapy on recurrent/metastatic disease. The widespread availability of combination checkpoint inhibitor therapy during the time course of this trial, however, meant that a proportion of patients who relapsed on the placebo arm did not crossover to pembrolizumab on trial and hopefully the investigators will capture this in further updates of the placebo arm.

These two anti-PD1 trials demonstrated the efficacy and safety of anti-PD1 in the adjuvant setting and as such became an accepted standard of care.

## Adjuvant Targeted Therapy

For patients with resected stage IIIA (metastasis > 1 mm), IIIB and IIIC melanoma (AJCCv7) harbouring the BRAF V600E or V600K mutations, the COMBI-AD trial demonstrated significantly improved RFS with combination BRAF/MEK inhibitors dabrafenib and trametinib compared with placebo [5, 12]. At preliminary analysis, the 3-year OS was 86% in the treatment group versus 77% in the placebo group (HR 0.57, 95% CI 0.42–0.79, *p*-value 0.0006) but did not cross the prespecified conservative *p*-value boundary. At 5-year follow-up, RFS was 52% in the treatment group and 36% in the placebo group (HR 0.51, 95% CI 0.42–0.61) but the number of events to trigger overall survival analysis had not yet been reached. Grade ≥ 3 adverse events occurred in 41% in the treatment group and 14% in the placebo group, with a discontinuation rate of 26% but there was no difference in the incidence of long-term adverse effects.

There are currently no head-to-head comparisons between immunotherapy and targeted therapy in the adjuvant setting and the difference in the observed RFS benefit between the three approved options is marginal. The choice of adjuvant therapy for patients with BRAF-mutant disease depends on an informed discussion between the oncologist and the patient, with acceptance of short-term adverse events with targeted therapy versus long-term toxicities associated with immunotherapy.

## Relapse During or After Adjuvant Therapy

There is a lack of prospective randomised studies to guide the management for patients who develop disease relapse during or after adjuvant therapy. The approach to further therapy in these patients is based on factors including type of adjuvant therapy received, BRAF mutation status, timing of relapse to adjuvant therapy and location of relapsed disease. Two recent retrospective studies have explored patterns of recurrence and subsequent management in patients who had undergone adjuvant immunotherapy and targeted therapy [13, 14].

## Patterns of Disease Relapse

With an understanding of the mechanism of these agents and clinical outcomes in the metastatic setting, it is not surprising to find that the majority of relapses on adjuvant immunotherapy occur within the first 12 months while on treatment [6, 7, 13], but relapse during the first 12 months on adjuvant targeted therapy is rare [5, 14, 15].

For patients who relapse with adjuvant anti-PD1, Owen et al. found that out of 147 patients, the median time to first relapse from starting treatment was 4.6 months. Most patients (*n* = 104, 76%) relapsed on treatment at a median of 3.2 months. For the 32 patients who relapsed off treatment, only 10 had managed to complete the full 12 months of treatment, and the median time to relapse from treatment cessation was 5.5 months. Initial recurrence was distant in 56% of cases.

In the second retrospective study, Bhave et al. identified 85 patients who had relapsed disease following adjuvant targeted therapy with a longer median time to recurrence of 17.7 months, and initial recurrence was distant in 66% of patients.

## Local Treatment for Relapsed Disease

Most patients with locoregional or resectable distant relapse in both retrospective cohorts underwent surgical resection, but subsequent relapse rates were high. For the cohort with adjuvant anti-PD1 therapy, 27/48 (56%) patients with resected locoregional disease developed further recurrence after a median follow-up of 8.3 months and 17/25 (68%) who underwent definitive local therapy to distant recurrence without systemic therapy developed further recurrence after a median follow-up of 16.7 months [13]. For the cohort with adjuvant targeted therapy, 14/23 (61%) patients with resected locoregional recurrence, and a total of 22/26 (85%) who underwent surgery without systemic therapy developed subsequent recurrence [14].

Both studies suggested that locoregional or resectable distant relapse following adjuvant systemic therapy is unlikely to be cured by surgery alone and that further systemic therapy is likely needed. However, there is currently insufficient data to understand the effectiveness or determine the duration required of this ‘second’ adjuvant systemic treatment.

One could speculate that solitary or oligo-relapses which occur late following adjuvant therapy may have more biologically favourable characteristics and be more suitable for aggressive local therapy with either surgery or stereotactic radiotherapy, but at this stage, there is no data to guide this speculation.

## Systemic Therapy Following Immunotherapy

Owen et al. grouped patients who underwent systemic therapy at first recurrence following anti-PD1 therapy into those who received ipilimumab-based therapy (with or without anti-PD1), BRAF/MEK inhibitor, further anti-PD1 alone or anti-PD1 with an investigational agent [13].

Targeted therapy with a BRAF and MEK inhibitor and ipilimumab-based therapy produced response rates of 82% (27/33) and 26% (10/38) respectively, similar to those observed in the treatment-naïve metastatic setting [16–18]. Ipilimumab with or without anti-PD1 seemed to demonstrate similar rates of response in this cohort, although data in the metastatic setting suggest that combination (anti CTLA4 + PD1) therapy is more effective than ipilimumab alone after anti-PD1 resistance. A retrospective cohort study reported a higher response rate (31% versus 13%) and median overall survival (20.4 months versus 8.8 months) for combination therapy over ipilimumab alone [19]. This data is supported by a phase II prospective study of 70 patients who had progressed on immunotherapy, including 13 (19%) from the adjuvant setting, with a similar response rate of 29% [20].

There was no response in the six patients who were treated with anti-PD1 monotherapy following relapse on adjuvant anti-PD1, although there was response in 2/5 (40%) patients who recurred off adjuvant anti-PD1 therapy with relapse at 5.6 months and 13.5 months. This confirms that similar to other classes of systemic therapies, a longer treatment-free interval in immunotherapy predicts a better outcome upon rechallenge; however, the exact time interval from cessation of adjuvant immunotherapy for which rechallenge with anti-PD1 would be efficacious remains undefined.

Patients with BRAF wild-type disease who relapse on or after adjuvant anti-PD1 therapy should therefore be considered for ipilimumab, combination with ipilimumab/nivolumab or novel immunotherapy combination trials as further monotherapy is likely to be ineffective. For patients with BRAF-mutant disease, consideration should be given to the options of targeted therapy or immunotherapy. Targeted therapy has a much better chance at achieving rapid disease control, such as in cases with high symptomatic disease burden, but may lead to fewer durable responses and long-term survival [16, 21]. Combination immunotherapy is a reasonable therapeutic option for select BRAF-mutant patients who have relapsed on adjuvant anti-PD1 but given the established anti-PD1 resistance, the efficacy of combination therapy is significantly less than in the treatment-naïve setting [19].

## Systemic Therapy Following Targeted Therapy

For patients with BRAF-mutant disease, Bhave et al. found that anti-PD1 therapy remained effective at first recurrence following adjuvant targeted therapy [14]. Response rates to anti-PD1-based therapy (with or without a trial agent) and ipilimumab/nivolumab combination therapy were 63% (12/19) and 62% (8/13) respectively. None of the five patients who developed relapse during adjuvant targeted therapy responded to treatment with subsequent immunotherapy, suggesting that this is a subset of patients with particularly aggressive disease with very unfavourable biology and broad treatment resistance.

While the sample size was small, it suggests the efficacy of immunotherapy might not be diminished by prior treatment with targeted therapy, at least for patients who relapse after completing targeted therapy, but this needs to be confirmed in a larger cohort given that in the metastatic setting, the response rate of single agent anti-PD1 fell from 30–40% to around 25% following progression on targeted therapy [22].

The response rate for re-treatment with targeted therapy after completing 12 months of adjuvant targeted therapy was 25% (4/16). This is congruent with observations in the metastatic setting, where the response rate to targeted therapy re-treatment has been documented between 27 and 43% [23–25], although most of the patients in these studies were treated with immunotherapy between the two phases of targeted therapy. The minimal interval from completion of adjuvant targeted therapy for re-treatment remains to be defined, but presumably the longer the more likely to be effective.

Given the superior response rate and potential for a more durable response, immunotherapy should be considered first when there is disease relapse on or after adjuvant targeted therapy. Combination with ipilimumab/nivolumab should be considered given the differential benefit of combination therapy over nivolumab alone was greater in patients with BRAF-mutant disease [26].

## Neoadjuvant Therapy

There are certainly significant theoretical advantages of neoadjuvant therapy over adjuvant therapy. Preclinical evidence showed that neoadjuvant immunotherapy, while macroscopic disease is still present, generates a richer and more robust clonal T cell response [27]. Neoadjuvant therapy also offers the advantage of in vivo sensitivity evaluation with pathological response showing great promise as an effective early surrogate for long-term outcomes. This is especially the case with immunotherapy. The quality of pathological response can then logically be used to risk-stratify and potentially escalate or de-escalate subsequent adjuvant therapy. However, at the present time, due to relatively small patient numbers with heterogenous treatment duration in uncontrolled trials with limited follow-up, neoadjuvant therapy is not considered standard of care and should only be offered in the context of a clinical trial. The International Neoadjuvant Melanoma Consortium (INMC) was established to develop recommendations for standardised trial design [28].

The INMC published a pooled analysis on from six neoadjuvant trials which included a total of 192 patients, of which 104 received ipilimumab/nivolumab, 37 single anti-PD1 and 51 targeted therapy [29]. Pathological complete response (pCR) was found in 43%, 20% and 47% of patients treated with combination immunotherapy, single anti-PD1 and targeted therapy respectively. Patients with pCR, near pCR or partial response with immunotherapy demonstrated a 2-year RFS of 96%, and patients with pCR with targeted therapy had a 2-year RFS of 79%. Accordingly, patients with no pathological response (pNR) had the poorest RFS of 30% at 2 years. Pathological partial response (pPR) with immunotherapy had a similar RFS to those with pCR; conversely, pPR with targeted therapy showed similar outcomes to those with pNR. These results suggested employing pathological response as a surrogate endpoint in future neoadjuvant immunotherapy trials.

There are no studies investigating patterns of relapse and management following neoadjuvant therapy. All current neoadjuvant trials included an adjuvant phase of treatment and management of relapse should consider the principles that have been discussed. However, neoadjuvant treatment may open the opportunity to analyse for mechanisms of resistance when a pathological response is not achieved, which could lead to personalizing adjuvant treatment [29].

With these potential advantages, neoadjuvant immunotherapy may eventually supersede adjuvant immunotherapy for a selection of patients. Studies are under way to assess neoadjuvant against adjuvant therapy, such as the NADINA trial (NCT04949113), in which patients who achieve a pathological response to neoadjuvant ipilimumab/nivolumab do not continue with adjuvant nivolumab. In future, the challenge of selecting appropriate the next line of therapy may be brought forward to the adjuvant phase in patients with a poor or no pathological response to neoadjuvant treatment, rather than on disease relapse.

## Future Considerations

The optimal duration of adjuvant treatment remains unclear, and all clinical trial protocols arbitrarily designated a course of 12 months. Data from neoadjuvant trials showed a high rate of pathological complete, or near-complete, response after only 3–6 weeks of immunotherapy [30, 31]. Similar patterns are seen in advanced melanoma where most responses are seen in the first 6 months of therapy [17, 18, 32]. This suggests that a significant proportion of patients may be suitable for a shorter duration of therapy that could be evaluated in future adjuvant clinical trials which may have significant health economic implications. The incidence of early relapse on cessation of targeted therapy is a more challenging scenario given that the 12 months of adjuvant therapy coincides with the average PFS benefit that we see in all the targeted therapy trials in advanced disease, potentially suggesting that a longer duration of therapy may not overcome targeted therapy resistance [21, 33, 34].

Despite intensive research searching for a biomarker for response to therapy, there is currently no biomarker to assist clinicians in the optimal choice of adjuvant therapy. The development of reliable, non-invasive liquid biomarkers could be advantageous for patients who have an identifiable truncal mutation, such as BRAF, NRAS or TERT promoter mutations, as guidance on adjuvant treatment selection and duration, as well as identifying low-risk patients who might not require any treatment [35]. In a clinical validation study, BRAF V600E-mutant circulating tumour DNA (ctDNA) levels at baseline and on treatment appeared to correlate with clinical outcomes in patients receiving targeted therapy for metastatic melanoma [36]. In patients with stage II/III melanoma, detection of both preoperative and postoperative ctDNA has been shown to be a predictor for disease relapse and poorer outcomes [37–39]. As an example, in the postoperative setting, a study found that mutated ctDNA was detected in 15/132 (11%) of BRAF mutant and 4/29 (14%) of NRAS mutant patient samples. Five-year OS was significantly lower for those with detectable ctDNA at 33% compared to 65%. However, 26% of patients with undetectable ctDNA developed disease relapse by 1 year, with a sensitivity for predicting relapse of 18% and negative predictive value of 51% [39]. While there was a strong correlation with outcomes, ctDNA is currently insufficient for identifying patients who can be spared the toxicity and cost of adjuvant treatment or interval testing during adjuvant treatment as a marker of treatment response. To be considered for widespread clinical application, it will require significant improvement in sensitivity, cost reduction and technique standardization [35].

Clinical trials are currently underway incorporating ctDNA in the clinical decision pathways for patients in the adjuvant setting, such as in the DETECTION study (NCT04901988), where patients with resected stage IIB/C disease are randomised to standard of care observation versus treatment with adjuvant nivolumab according to ctDNA results.

The ongoing phase III KEYNOTE-716 trial is exploring adjuvant pembrolizumab in resected stage IIB/C disease [40]. Interim analysis showed significant RFS benefit of pembrolizumab compared with placebo (HR 0.65, 95% CI 0.46–0.92). The melanoma-specific survival for these patients with these thicker, ulcerated primary tumours is comparable to, or less favourable than, those with stage IIIA and IIIB disease with thinner primaries and micro-metastatic nodal disease [1]. Potentially this will significantly increase our adjuvant treatment population, with impact on treatment choices for those who subsequently relapse.

Talimogene laherparepvec (T-VEC) is a herpes simplex virus type 1-based intralesional oncolytic therapy, for which a randomised phase II trial of neoadjuvant T-VEC with surgery versus surgery alone for resectable stage IIIB-IVM1a disease showed a significant 25% reduction in 2-year RFS (HR 0.75, 80% CI 0.58–0.96) [41]. While it requires further investigation before consideration for routine use in the neoadjuvant setting, it raises the possibility of exploring T-VEC and other intralesional therapies as novel classes of treatment for relapsed disease that is accessible for direct injection.

Novel systemic treatment combinations are being explored for metastatic melanoma in phase III studies, including immunotherapy combinations with relatlimab, a lymphocyte-activation gene 3 antibody and lenvatinib, an oral multikinase inhibitor (NCT03820986). The recently published RELATIVITY-047 trial showed a significant benefit in progression-free survival with relatlimab/nivolumab over nivolumab alone, with much lower toxicity than we are used to seeing with ipilimumab/nivolumab [42]. These studies excluded patients who relapsed on or within 6 months of adjuvant therapy and as such there will still be a paucity of data on the efficacy of these novel regimens in patients who are exposed to adjuvant therapy. They may, however, become appropriate alternatives on disease relapse especially if a patient is deemed unsuitable for combination therapy with ipilimumab or targeted therapy is not an option.

## Conclusions

The landscape for adjuvant therapy in resected stage III melanoma has changed significantly over the past decade, with the demonstration of efficacy with immunotherapy and targeted therapy. We have now also seen early signal for efficacy of adjuvant immunotherapy in resected stage IIB/C disease. This means that increasingly stage III and higher risk stage II patients will have been exposed to some form of adjuvant therapy prior to relapse.

The current effort in taking neoadjuvant therapy into larger, standardised trials has the potential to provide not only a new treatment paradigm for locally advanced melanoma, but translational information about mechanisms of response and resistance, and may play a role in accelerating the development of novel therapies by giving an early read-out in the form of pathological response rates. Predictive biomarkers of relapse, such as ctDNA, may also become more refined in the future to be able to practically guide selection of and determine effectiveness of adjuvant therapy.

While cure is the intent, relapse on or after adjuvant and neoadjuvant therapy will unfortunately continue to increase in absolute numbers as more and more patients receive treatment in these settings. The optimal management for these patients is yet to be defined in the literature. The results of the pivotal adjuvant trials of CheckMate 238, COMBI-AD and KEYNOTE-054 are practice changing, but the outcomes of the substantial cohorts of patients who progressed despite adjuvant treatment are largely unknown.

The existing, small retrospective cohort studies are of limited utility in guiding decision-making. Treatment that the patient has not yet been exposed to appears to be most appropriate on first principles, but efficacy is uncertain. Rechallenge with the agent used in the adjuvant setting might be reasonable with a longer interval to disease relapse. Patients with BRAF wild-type disease who relapse early during adjuvant treatment are in particular need of effective subsequent treatment options.

There is therefore an urgent need for widespread, uniform and robust data to be collected on patients who develop disease relapse. These need to include not only the pattern of relapse with respect to timing and location, but management strategies, treatment modalities and, importantly, following outcomes through to subsequent relapse or sustained disease control. Incorporating this into the design of future prospective adjuvant trials can be one strategy to build this body of data.

Future first-line systemic therapy trials also need to consider this patient population as existing studies only include those who have been off therapy for over 6 months. This could come in the form of dedicated studies for those who relapse on or soon after adjuvant systemic therapy, or allowance for this cohort to be a capped proportion within proposed studies, and would be of vital importance for oncologists in selecting the most appropriate treatment options for their patients.
